# Risk of cardiovascular mortality in patients with gastric cancer

**DOI:** 10.1371/journal.pone.0335989

**Published:** 2025-11-07

**Authors:** Tian Zhang, Lingling Guan, Xiaomeng Xie, Juecai Chen, Dongjing Ni

**Affiliations:** 1 Department of Pathology, Shiyan People’s Hospital of Bao’an District, Shenzhen, Guangdong, China; 2 Department of Gastroenterology, The Fifth Affiliated Hospital of Jinan University, Heyuan, Guangdong, China; 3 Department of Hematology, The First Affiliated Hospital of Chengdu Medical College, Chengdu, China; 4 Department of Gastroenterology, The Fifth Hospital of Jinan University, Heyuan, Guangdong, China; The University of the West Indies, JAMAICA

## Abstract

**Purpose:**

To assess the excess cardiovascular disease (CVD) mortality risk in patients with gastric cancer (GC) compared with the general population.

**Methods:**

Using data from the National Cancer Institute’s Surveillance, Epidemiology, and End Results (SEER) program, we conducted a retrospective, population-based cohort study of 41,083 GC patients ≥30 years old between January 1, 1990, and December 31, 2021. A reference cohort from the corresponding general population was also included. Incidence rate ratios (IRRs) for CVD mortality were estimated using Poisson regression.

**Results:**

GC patients were associated with a higher risk of CVD mortality relative to the general population (IRR: 1.46; 95% CI: 1.40–1.52). The relative risk was highest among younger patients aged 30–39 years (IRR: 7.69; 95% CI: 2.48–23.86). Cardiovascular mortality risk peaked during the first month after cancer diagnosis (IRR: 11.52; 95% CI: 9.97–13.31). The relationship between GC and CVD mortality varied according to demographic and clinical characteristics.

**Conclusions:**

These population-based findings support early cardiac assessment and comprehensive risk stratification in GC care. Prospective studies with richer confounder data are needed to test whether these strategies improve outcomes.

## Introduction

Gastric cancer (GC) ranks fifth worldwide for both incidence and cancer-related mortality, accounting for approximately 968,350 new cases and 659,853 deaths in 2022 [1]. Although the incidence has declined over recent decades, GC remains a substantial public health burden. Despite advances in multimodal therapies (surgery, chemotherapy, radiotherapy, and targeted agents), GC maintains a poor prognosis with a 5-year survival rate of approximately 25% [2].

Cardiovascular disease (CVD) accounts for approximately 5.9% of all deaths in GC patients, highlighting its contribution to overall mortality [3]. GC and CVD share risk factors, including hypertension, obesity, diabetes, smoking, dyslipidemia, and chronic inflammation, which have been associated with higher baseline rates of cardiovascular events and mortality among GC patients [4,5]. Moreover, GC treatments can be cardiotoxic. Radiotherapy (especially with thoracic exposure), fluorouracil-based chemotherapy, anthracyclines, and targeted therapies (e.g., TKIs) are linked to heart failure, arrhythmias, QT prolongation, and thromboembolic events, and may be associated with higher CVD-related mortality [6–12]. In addition, certain subgroups of GC patients are related to an increased risk of CVD-related mortality. This pattern may reflect the cumulative cardiotoxicity associated with cancer treatments such as chemotherapy and radiotherapy, together with pre-existing cardiovascular risk factors including hypertension, diabetes mellitus, obesity, dyslipidemia, and smoking [4,13,14]. In addition, the inflammatory response, triggered by both the malignancy itself and its associated treatments, is implicated in the development and progression of CVD [15,16]. These overlapping risks support systematic cardiovascular risk assessment and the incorporation of cardioprotective strategies into multidisciplinary treatment planning for GC patients [17]. Meanwhile, psychological stress associated with a GC diagnosis has been linked to higher CVD mortality risk via mechanisms involving neuroendocrine dysregulation, excessive sympathetic nervous system activation, and systemic inflammation [18]. However, comprehensive studies providing population-based estimates of CVD mortality risk in GC relative to the general population remain limited.

Leveraging the population-based Surveillance, Epidemiology, and End Results (SEER) registry, this study quantifies CVD mortality risk in GC patients relative to the general population through incidence rate ratio (IRR) analysis, while characterizing long-term prognostic trajectories.

## Materials and methods

### Patients and methods

A retrospective cohort study was performed using data from the SEER database, including 66,581 patients with a pathological diagnosis of primary GC between January 1, 1990, and December 31, 2021. Person-time from the general population (55,421,308 person-years) served as the reference group to estimate associations between GC patients and general population.

**Fig 1:**
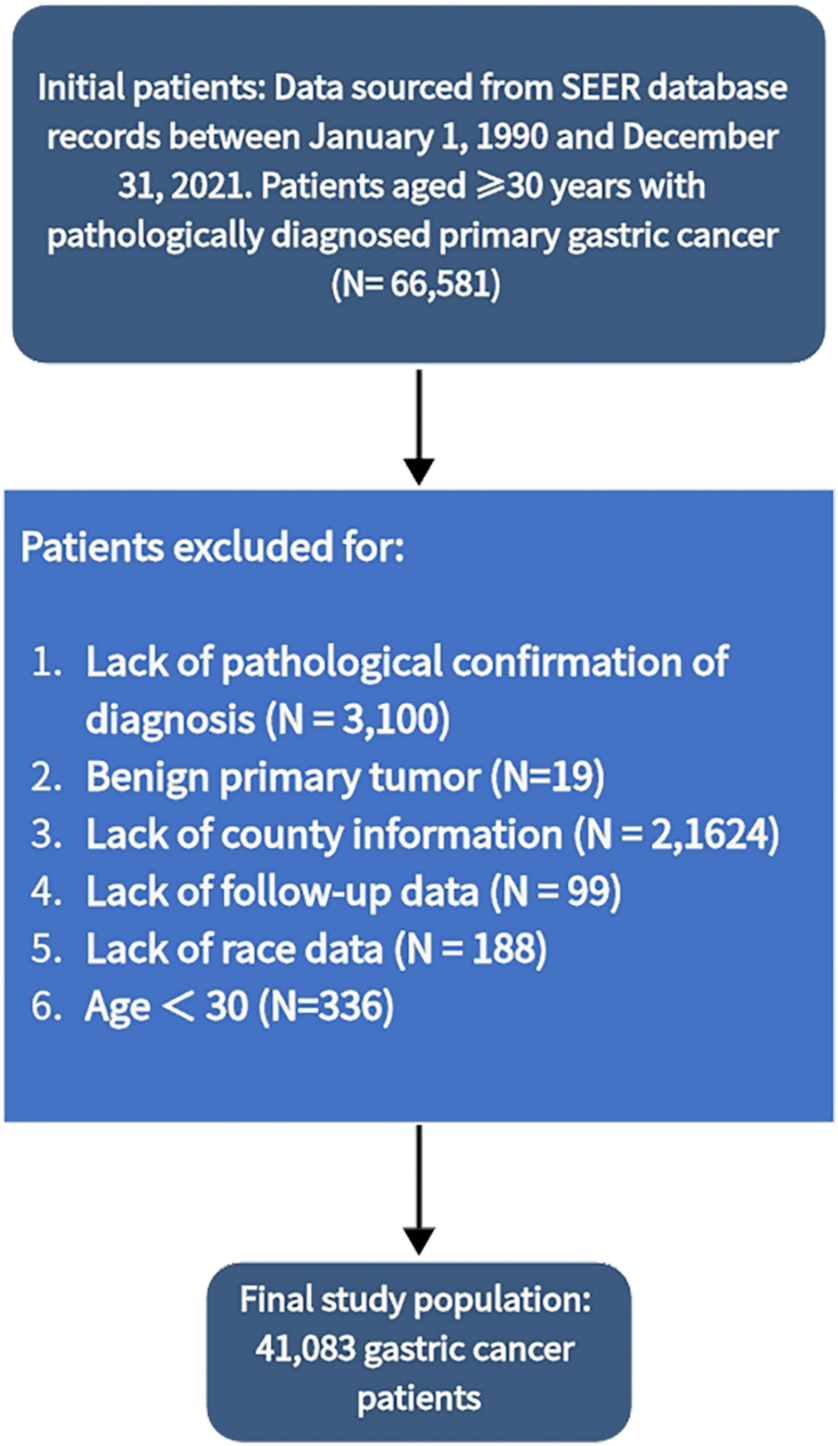
Flowchart.

Patients were excluded if they met any of the following criteria: lack of pathological confirmation (N = 3,107); benign primary tumor (N = 19); missing county information (N = 21,739); lack of follow-up data (N = 100); missing race data (N = 197); or age < 30 years (N = 336). The final analytic cohort included 41,083 patients with confirmed GC (Fig 1).

### Confirmation of CVD-related deaths and follow-up

The primary outcome was CVD mortality. CVD-related deaths were identified using International Classification of Diseases (ICD) codes from the SEER Cause of Death Recode and classified as heart disease, cerebrovascular disease, or other cardiovascular conditions [19,20]. Follow-up was ascertained through SEER registry procedures, including routine linkages to state and national mortality files and other administrative sources; mortality information for the reference general-population cohort was derived from population-based vital statistics for the same calendar period. No direct participant re-contact was performed in this study [20].

### Variables

Demographic data for GC patients and the general population were retrieved from the SEER database and the U.S. Census Bureau’s Population Estimates Program. These data included age, calendar year at follow-up or cancer diagnosis (1990–2021), race (White, Black, or other), sex (male or female), and county type (counties in metropolitan areas with populations >1,000,000; 250,000–1,000,000; < 250,000; non-metropolitan not adjacent to a metropolitan area; non-metropolitan adjacent to a metropolitan area). Age at follow-up, a key predictor of CVD mortality risk [21], was categorized into 10-year intervals (30–39, 40–49, 50–59, 60–69, 70–79, and ≥80 years) to improve comparability and reduce age-related confounding. Clinical variables for GC patients were also sourced from the SEER database, including laterality, tumor stage, histology, grade, surgery (yes, no, and unknown), radiotherapy (yes, no/unknown), and chemotherapy (yes, no/unknown). Missing data were coded as “unknown.” Baseline characteristics of GC patients and the general population are shown in Table 1.

**Table 1:** Baselines of GC patients and the U.S. population: a population-based cohort study in the U.S., 1990-2021.

	Gastric Patients	Population
	Per 100 PYs (%)	Per 100 PYs (%)
**Total**	1,200 (100.00)	55,421,308 (100.00)
**Calendar year at follow-up**		
1990–1992	26 (2.19)	4,181,956 (7.55)
1993–1995	52 (4.33)	4,438,414 (8.01)
1996–1998	68 (5.66)	4,656,877 (8.40)
1999–2001	84 (7.03)	4,879,776 (8.80)
2002–2004	103 (8.55)	5,070,143 (9.15)
2005–2007	122 (10.19)	5,239,649 (9.45)
2008–2010	141 (11.73)	5,423,250 (9.79)
2011–2013	162 (13.52)	5,618,647 (10.14)
2014–2016	182 (15.14)	5,813,843 (10.49)
2017–2021	260 (21.64)	10,098,755 (18.22)
**Age groups at follow up, year**		
30–39	22 (1.81)	13,525,444 (24.40)
40–49	87 (7.23)	13,104,984 (23.65)
50–59	198 (16.51)	11,368,846 (20.51)
60–69	305 (25.44)	8,574,909 (15.47)
70–79	330 (27.48)	5,582,078 (10.07)
80–89	258 (21.52)	3,265,049 (5.89)
**Sex**		
Male	499 (41.61)	28,880,074 (52.11)
Female	701 (58.39)	26,541,236 (47.89)
**Race**		
White	819 (68.22)	45,812,876 (82.66)
Black	131 (10.88)	6,465,842 (11.67)
Other	251 (20.90)	3,142,593 (5.67)
**County**		
Counties in metropolitan areas of larger than 1 million population	642 (53.53)	30,226,526 (54.54)
Counties in metropolitan areas of 250,000–1 million population	330 (27.46)	11,547,770 (20.84)
Counties in metropolitan areas of less than 250 thousand population	78 (6.46)	4,992,200 (9.01)
Nonmetropolitan counties not adjacent to a metropolitan area	74 (6.15)	2,954,069 (5.33)
Nonmetropolitan counties adjacent to a metropolitan area	77 (6.40)	5,700,744 (10.29)
**Income**		
Low-income	146 (12.14)	
Middle-income	269 (22.38)	
High-income	786 (65.48)	
**Cohabitation**		
No	374 (31.16)	
Yes	766 (63.81)	
Unknown	60 (5.03)	
**Laterality**		
Cardia	306 (25.50)	
Fundus-of-gastric	64 (5.33)	
Body-of-gastric	110 (9.14)	
Gastric-antrum	229 (19.08)	
Pylorus	31 (2.60)	
Curvature-of-gastric	137 (11.44)	
Greater-curvature-of-gastric	77 (6.44)	
Overlapping-lesion-of-gastric	63 (5.27)	
Gastric	183 (15.21)	
**Time since diagnosis**		
0 to <1 month	32 (2.68)	
1 to <6 month	124 (10.30)	
6 to <12 month	115 (9.56)	
1 to <2 years	162 (13.46)	
2 to <5 years	296 (24.62)	
5 to <10 years	267 (22.27)	
≥ 10 years	205 (17.09)	
**Histology**		
Adenocarcinoma	706 (58.85)	
Signet-ring-cell-carcinoma	148 (12.34)	
Mucinous-carcinoma	24 (1.99)	
Others	322 (26.82)	
**Tumor grade**		
Well differentiated	116 (9.63)	
Moderately differentiated	277 (23.10)	
Poorly differentiated	468 (38.99)	
Undifferentiated	28 (2.35)	
Unknown	311 (25.94)	
**Tumor stage**		
Local	606 (50.52)	
Regional	381 (31.78)	
Distant	138 (11.51)	
Unknown	74 (6.19)	
**Surgery**		
No	221 (18.38)	
Yes	975 (81.24)	
Unknown	4.5 (0.37)	
**Radiation**		
No/unknown	921 (76.75)	
Yes	279 (23.25)	

CI, confidence interval; PYs, person-years.

### Statistical by clinical and demographic factors

Poisson regression was used to estimate the IRRs and 95% confidence intervals (95% CIs) for the association between GC patients and CVD mortality relative to the general population, adjusting for age at follow-up, race, sex, county type, and calendar year.

We characterized the association by age group and by time since diagnosis. Subtype-specific IRRs were calculated for heart disease (SEER code 50060), cerebrovascular diseases (50080), and other cardiovascular conditions (50070, 50090, 50100, 50110) to report cause-specific associations [22].

Statistical analyses were performed using Stata version 16.0 (StataCorp). Two-sided p-values <0.05 were considered statistically significant for the association estimates.

### Ethics statement

This study uses publicly available, de-identified data from the Surveillance, Epidemiology, and End Results (SEER) database. Because SEER database does not include personally identifiable information, the study is exempt from Institutional Review Board (IRB) review, and informed consent is not required.

## Results

### Survival characteristics

A total of 41,083 GC patients were diagnosed between 1990 and 2021, with 2,288 deaths attributed to CVD (mortality rate: 1.91 per 100 person-years). The median follow-up was 11 months (interquartile range, 3−38 months). In comparison, the general population had 27,870,722 CVD deaths (mortality rate: 0.50 per 100 person-years). Total person-time was 1,200 person-years in the GC cohort and 55,421,308 person-years in the general population (Table 1). Overall, GC patients were associated with higher risk of CVD mortality relative to the general population (IRR 1.46, 95% CI 1.40–1.52; Table 2).

**Table 2:** Incidence rate ratios (IRRs) of cardiovascular mortality in GC patients compared with the general population: a population-based cohort study in the U.S., 1990-2021.

	Population N (MR)	Patients N (MR)	IRR (95% CI)^a^
Overall	27,870,722 (0.50)	2,288 (1.91)	1.46 (1.40–1.52)
Age groups at follow up, years			
30–39	256,771 (0.02)	3 (0.14)	7.69 (2.48–23.86)
40–49	851,762 (0.06)	17 (0.20)	3.03 (1.88–4.88)
50–59	2,007,231 (0.18)	99 (0.50)	2.79 (2.29–3.40)
60–69	3,700,723 (0.43)	289 (0.95)	2.23 (1.99–2.51)
70–79	6,346,636 (1.14)	584 (1.77)	1.66 (1.53–1.80)
80+	14,707,599 (4.50)	1,296 (5.02)	1.25 (1.19–1.32)
P for interaction^b^			<0.001

Cl, confidence interval; IRR, incidence rate ratio; MR, mortality rate per 100 person-years; N, number of deaths. Data were obtained from the SEER database between 1990 and 2021. Poisson regression was used for risk estimation.

^a^IRRs were adjusted for age at follow-up (30–39 years, every 10years thereafter, and ≥ 80years).sex (female or male), race (white, Black, or other), county (counties in metropolitan areas with a population larger than 1 million, counties in metropolitan areas with a population of 250,000–1,000,000, counties in metropolitan areas with a population of less than250,000,non-metropolitan counties not adjacent to a metropolitan area, or non-metropolitan counties adjacent to a metropolitan area), and calendar year at follow-up (1990–1992,1993–1995,1996–1998,1999–2001,2002–2004,2005–2007,2008–2010,2011–2013,2014–2016,2017–2021).

^b^We added an interaction between GC and age at follow-up (30–39 years, every10 years thereafter, and ≥ 80years) and reported the significance level of the term as p for interaction.

### Age at follow-up and time elapsed since GC diagnosis

**Fig 2:**
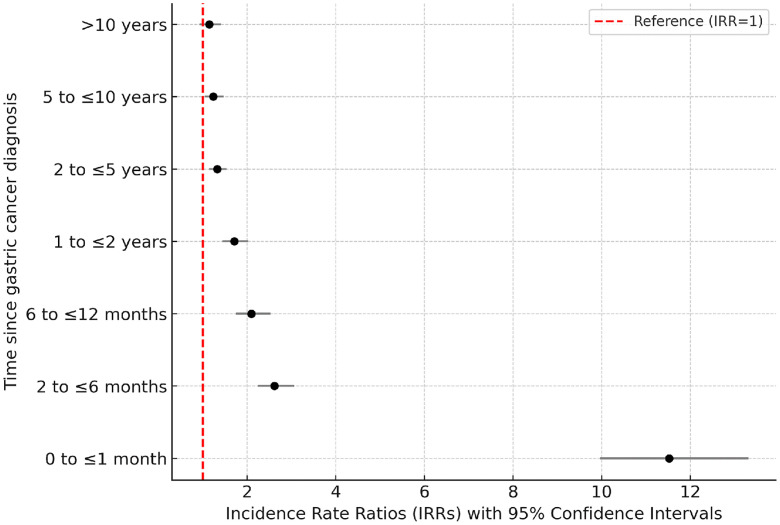
IRRs of cardiovascular mortality by time since cancer diagnosis, comparing GC patients with the general population. Incidence rate ratios (IRRs) of cardiovascular mortality by time since GC diagnosis, comparing patients with the general population. Forest plot shows IRRs with 95% confidence intervals across different follow-up intervals. The vertical dashed line represents the reference value (IRR = 1). IRR, incidence rate ratio; CI, confidence interval; GC, gastric cancer.

Patients aged 30–39 years at follow-up were linked to the highest risk of CVD mortality (IRR 7.69, 95% CI 2.48–23.86; Table 2). From 40 to 80, IRRs decreased from 3.03 to 1.25, indicating a gradual reduction in relative risk with increasing age (Table 2). The risk of CVD mortality was highest during the first month after diagnosis (IRR 11.52, 95% CI 9.97–13.31; Fig 2; S1 Table). Although this risk declined after the first month, it remained statistically significant thereafter.

### Competing risks of cancer-related death analysis

A competing-risks cumulative incidence analysis was also conducted to account for cancer-related deaths as a competing event. The results were consistent with Poisson regression showing that cancer-related mortality risk (cumulative incidence) rose most rapidly early after diagnosis, while CVD mortality showed the steepest early increase within the first month after diagnosis. Over longer follow-up, both CVD and other-cause deaths accumulated, indicating the increasing contribution of non-cancer causes among long-term survivors (S1 Fig).

By 20 years after diagnosis, the cumulative incidence of CVD mortality was 7.6%, exceeding that of GC (5.2%) and other causes (3.2%) (S1 Fig).

### Subgroup analysis

Prespecified subgroup analysis of CVD subtypes are linked to higher CVD mortality risk in GC patients than in the general population: heart disease (IRR 1.47, 95% CI 1.40–1.52), cerebrovascular disease (IRR 1.46, 95% CI 1.32–1.61) relative to the general population (Table 3). And we did not interpret estimates with wide confidence intervals.

**Table 3:** Incidence rate ratios (IRRs) of cause-specific cardiovascular mortality in GC patients compared with the general population, U.S., 1990–2021.

	Population N (MR)	Patients N (MR)	IRR (95% CI)^a^
**Diseases of heart**	21,362,048 (0.39)	1,736 (1.45)	1.47 (1.40–1.52)
**Cerebrovascular diseases**	4,684,652 (0.09)	404 (0.34)	1.46 (1.32–1.61)
**Other cardiovascular diseases**	1,809,541 (0.03)	148 (0.12)	1.38 (1.17–1.62)

Cl, confidence interval; IRR, incidence rate ratio; MR, mortality rate per 100 person-years; N, number of deaths. Data were obtained from the SEER database between 1990 and 2021. Poisson regression was used for risk estimation.

a. IRRs were adjusted for age at follow-up (30–39 years, every 10years thereafter, and ≥ 80years).sex (female or male), race (white, Black, or other), county (counties in metropolitan areas with a population larger than 1 million, counties in metropolitan areas with a population of 250,000–1,000,000, counties in metropolitan areas with a population of less than250,000,non-metropolitan counties not adjacent to a metropolitan area, or non-metropolitan counties adjacent to a metropolitan area), and calendar year at follow-up(1990–1992,1993–1995,1996–1998,1999–2001,2002–2004,2005–2007,2008–2010,2011–2013,2014–2016,2017–2021).

### Demographic and clinical characteristics

A stronger association with CVD mortality risk was observed among GC patients who were American Indian/Alaska Native or Asian/Pacific Islander descent (IRR 1.72, 95% CI 1.56–1.91), as well as among those diagnosed in 1990−1992 (IRR 2.27, 95% CI 1.88–2.74). Higher risks were also observed in patients with distant-stage disease(IRR 2. 30, 95% CI 2. 04−2. 59), those who received radiotherapy (IRR 1. 45, 95% CI 1. 31−1. 60), those who did not undergo surgery (IRR 2. 52, 95% CI 2.4235−2. 71), and those with mucinous carcinoma histology(IRR 1. 68, 95% CI 1. 31−2. 17) (Tables 4 and 5).

**Table 4:** Incidence rate ratios (IRRs) of cardiovascular mortality in GC patients compared with the general population, stratified by demographic characteristics, U.S., 1990–2021.

	Patients N (MR)	Population N (MR)	IRR (95% CI) ^a^
**Sex**			
Male	913 (1.83)	14311338 (0.50)	1.52 (1.43–1.62)
Female	1375 (1.96)	13559384 (0.51)	1.42 (1.35–1.50)
P for interaction	.		0.103
**Race**			
White	1685 (2.06)	24023668 (0.52)	1.43 (1.36–1.50)
Black	228 (1.75)	3244481 (0.50)	1.32 (1.16–1.50)
Other(American Indian/AK Native, Asian/Pacific Islander)	375 (1.50)	602573 (0.19)	1.72(1.56–1.91)
P for interaction			0.001
**County**			
Counties in metropolitan areas of larger than 1 million population	1123 (1.75)	13792302 (0.46)	1.42 (1.34–1.51)
Counties in metropolitan areas of 250,000–1 million population	676 (2.05)	5804016 (0.50)	1.57 (1.46–1.70)
Counties in metropolitan areas of less than 250 thousand population	144 (1.86)	2735312 (0.55)	1.38 (1.17–1.62)
Nonmetropolitan counties not adjacent to a metropolitan area	192 (2.60)	1904368 (0.64)	1.57 (1.36–1.81)
Nonmetropolitan counties adjacent to a metropolitan area	153 (1.99)	3634724 (0.64)	1.23 (1.05–1.44)
P for interaction			0.033
**Calendar year at follow-up**			
1990–1992	108 (4.11)	2723937 (0.65)	2.27 (1.88–2.74)
1993–1995	163 (3.13)	2817540 (0.63)	1.65 (1.42–1.93)
1996–1998	214 (3.15)	2811752 (0.60)	1.71 (1.49–1.95)
1999–2001	244 (2.89)	2794193 (0.57)	1.67 (1.47–1.89)
2002–2004	209 (2.04)	2670210 (0.53)	1.29 (1.13–1.48)
2005–2007	235 (1.92)	2473593 (0.47)	1.39 (1.22–1.57)
2008–2010	231 (1.64)	2353307 (0.43)	1.35 (1.19–1.54)
2011–2013	258 (1.59)	2347380 (0.42)	1.44 (1.27–1.63)
2014–2016	259 (1.42)	2459937 (0.42)	1.34 (1.19–1.52)
2017–2021	367 (1.41)	4418873 (0.44)	1.33 (1.20–1.47)
P for interaction^b^			<0.001

CI, confidence interval; IRR, incidence rate ratio; MR, mortality rate per 100 person-years; N, number of deaths. Data were obtained from the SEER database between 1990 and 2021. Poisson regression was used for risk estimation.

^a^IRRs were adjusted for age at follow-up (30–39 years, every 10years thereafter, and ≥ 80years), sex (female or male), race (white, Black, or other), county (counties in metropolitan areas with a population larger than 1 million, counties in metropolitan areas with a population of 250,000–1,000,000, counties in metropolitan areas with a population of less than250,000,non-metropolitan counties not adjacent to a metropolitan area, or non-metropolitan counties adjacent to a metropolitan area), and calendar year at follow-up (1990–1992,1993–1995,1996–1998,1999–2001,2002–2004,2005–2007,2008–2010,2011–2013,2014–2016,2017–2021).

^b^The interaction term of IRRs across demographic characteristics was added and the p-value for significance was reported.

**Table 5:** Incidence rate ratios (IRRs) of cardiovascular mortality in GC patients compared with the general population, stratified by clinical characteristics, U.S., 1990–2021.

	Patients N (MR)	IRR (95% CI) ^a^
**Histology**		
Adenocarcinoma	1615 (2.29)	1.51 (1.43–1.58)
Signet-ring cell carcinoma	213 (1.44)	1.57 (1.37–1.80)
Mucinous carcinoma	60 (2.51)	1.68 (1.31–2.17)
Others	400 (1.24)	1.23 (1.11–1.36)
P for interaction^b^		0.001
**Tumor stage**		
Local	1114 (1.84)	1.31 (1.23–1.39)
Regional	625 (1.64)	1.31 (1.21–1.41)
Distant	277 (2.01)	2.26 (2.01–2.54)
Unstaged	272 (3.66)	2.30 (2.04–2.59)
P for interaction^b^		<0.001
**Tumor grade**		
Well differentiated	192 (1.66)	1.31 (1.14–1.51)
Moderately differentiated	607 (2.19)	1.39 (1.28–1.50)
Poorly differentiated	878 (1.88)	1.45 (1.35–1.55)
Undifferentiated	49 (1.74)	1.33 (1.01–1.76)
Unknown	562 (1.81)	1.64 (1.51–1.78)
P for interaction^b^	.	0.018
**Chemotherapy**		
No/unknown	1793 (2.39)	1.51 (1.44–1.58)
Yes	495 (1.10)	1.30 (1.19–1.42)
P for interaction		0.004
**Radiotherapy**		
No/unknown	1899 (2.06)	1.46 (1.40–1.53)
Yes	389 (1.39)	1.45 (1.31–1.60)
P for difference^b^		0.886
**Surgery**		
No/unknown	787 (3.50)	2.52 (2.35–2.71)
Yes	1501 (1.54)	1.19 (1.13–1.26)
P for difference^b^		<0.001

Cl, confidence interval; IRR, incidence rate ratio; MR, mortality rate per 100 person-years; N, number of deaths. Data were obtained from the SEER database between 1990 and 2021. Poisson regression was used for risk estimation.

^a^IRRs were adjusted for age at follow-up (30–39 years, every 10years thereafter).sex (female or male), race (white, Black, or other), county (counties in metropolitan areas with a population larger than 1 million, counties in metropolitan areas with a population of 250,000–1,000,000, counties in metropolitan areas with a population of less than250,000,non-metropolitan counties not adjacent to a metropolitan area, or non-metropolitan counties adjacent to a metropolitan area), and calendar year at follow-up (1990–1992,1993–1995,1996–1998,1999–2001,2002–2004,2005–2007,2008–2010,2011–2013,2014–2016,2017–2021).

^b^The interaction term of IRRs across demographic characteristics was added and the p-value for significance was reported.

## Discussion

This study indicates that GC patients have an elevated risk of CVD mortality compared with the general population. The risk of CVD mortality is highest during the first month after diagnosis and among younger patients (aged 30–39 years), those with advanced-stage disease, patients who do not undergo surgery, and those who receive radiotherapy. Elevated risks are also observed across CVD subtypes, including heart disease, cerebrovascular disease, and other CVD.

Radiation exposure during GC radiotherapy is linked to inflammatory cascades and subsequent myocardial fibrosis, thereby increasing the risk of CVD mortality [23], which is consistent with our findings that GC patients who received radiotherapy demonstrate an elevated risk of CVD mortality. Furthermore, prior studies report that advanced cancer stage is associated with higher CVD mortality risk [24]. Our findings are consistent with these observations. This association may be driven by elevated systemic inflammation, tumor burden, and the cumulative cardiotoxic effects of aggressive treatment modalities [25].

We also find that GC patients who did not undergo surgery have a significantly higher risk of cardiovascular mortality compared with those patients who undergo surgical treatment. This pattern may reflect several factors, including advanced tumor stage or poor functional status rendering patients ineligible for surgery, as well as the use of more intensive systemic therapies, such as chemotherapy alone, without the benefit of postoperative recovery support [24]. This disparity may reflect the combined impact of advanced disease stage, comorbid cardiovascular risk, and treatment-related toxicity in nonsurgical patients [3].

Age is associated with CVD mortality risk in both GC patients and the general population [26]. Our study found that CVD-related mortality risk increased progressively with aging at follow-up in both GC patients and the general population (Table 2). Notably, the most pronounced association with CVD mortality risk was observed in the youngest age group (30–39 years). This elevated association may be attributed to psychological stress, cardiotoxic effects of chemo-radiotherapy, and underlying comorbidities, suggesting that younger patients may particularly benefit from tailored CVD management and early intervention strategies [27]. This is consistent with a recent study indicating that, compared to the general U.S. population, cancer survivors diagnosed at a younger age face a higher risk of death from heart disease [3].

Our study also found that the association with CVD mortality is the highest within the first month after the diagnosis of GC (IRR 11.52, 95% CI 9.97–13.31; Fig 2; S1 Table). This early peak is plausibly attributable to some reasons. First, acute psychological or physiological stress surrounding the time of diagnosis may trigger sympathetic activation and precipitate acute events such as stress-induced cardiomyopathy [23]. Second, GC falls within the thrombosis-prone spectrum of upper gastrointestinal malignancies. Cancer-related hypercoagulability and inflammation drive arterial and venous thromboembolism around the first month after diagnosis and thereby precipitate myocardial infarction, ischemic stroke, or pulmonary embolism [28]. Third, when surgery is undertaken within the first month after diagnosis, perioperative ischemia, blood loss, infection, and fluid shifts, superimposed on pre-existing cardiovascular vulnerability, substantially increase the risk of myocardial injury, infarction, and death [29]. Fourth, some first-line regimens (e.g., fluoropyrimidines) can cause acute cardiotoxicity within hours to days of administration, manifesting as coronary vasospasm, ischemia, or malignant arrhythmias [30]. Accordingly, during the initial month after diagnosis, multidisciplinary care and cardiovascular risk assessment should be intensified. Antithrombotic strategies should be implemented according to thrombosis risk. Perioperative screening and management for myocardial injury should be standardized. For fluoropyrimidine-containing regimens, cardiotoxicity alert-and-response pathways should be established. These measures aim to mitigate adverse CVD outcomes during this high-risk window. In our study, the median follow-up time is 11 months. Additionally, our findings indicate that the cardiovascular mortality risk is significantly elevated within the first month following GC diagnosis. This early spike in CVD mortality risk appears to be robust and clinically relevant finding despite the short follow-up period.

The major strength of our research is the population-based cohort design, which helps minimize both selection and recall biases. The large sample size enables investigation into the associations between the CVD mortality risk and age at follow-up, time elapsed since cancer diagnosis, demographic factors as well as clinical characteristics.

This study has several limitations. First, due to the structural constraints of the SEER database, we were unable to adjust for key CVD risk factors, including pre-existing conditions (e.g., hypertension, diabetes mellitus, dyslipidemia), lifestyle behaviors (e.g., smoking, alcohol consumption, physical inactivity), body mass index, and prior CVD. These factors are well-established predictors of CVD mortality and may differ between GC patients and the general population. Although our large sample size and subgroup analyses mitigate some bias, the inability to adjust for these factors introduces a risk of residual or unmeasured confounding, which could influence our findings. Second, the lack of detailed treatment data, such as chemotherapy regimens, cumulative doses, and radiation fields, limited our ability to perform dose–response analyses or explore the treatment-related cardiotoxicity pathway. Subgroup analyses based on available treatment modalities partially addressed this limitation. Third, we acknowledge the potential for immortal time bias. However, since patients in the non-surgery group often have more advanced stages of GC, which is associated with higher early mortality, including deaths from both cancer and CVD. In contrast, patients who undergo surgery typically present with earlier-stage disease, and have lower short-term mortality. Given that cardiovascular mortality is primarily concentrated within the first month post-diagnosis, the impact of immortal time bias on our subgroup comparisons is likely limited.

## Conclusion

These findings highlight the need for a multidimensional risk assessment framework and suggest that prioritizing cardiovascular surveillance and individualized interventions may be beneficial for GC patients.

## Supporting information

S1 Table Incidence rate ratios (IRRs) of cardiovascular mortality in GC patients by time since cancer diagnosis, compared with the general population. Abbreviations: CI, confidence interval; IRR, incidence-rate ratio; MR, mortality rate per 100 person-years; N, number of deaths. ^a^ IRRs were adjusted for age at follow-up (30–39 years, every 10years thereafter, and ≥80 years).sex (female or male), race (white, Black, or other), county (counties in metropolitan areas with a population larger than 1 million, counties in metropolitan areas with a population of 250,000–1,000,000, counties in metropolitan areas with a population of less than250,000,non-metropolitan counties not adjacent to a metropolitan area, or non-metropolitan counties adjacent to a metropolitan area), and calendar year at follow-up (1990–1992,1993–1995,1996–1998,1999–2001,2002–2004,2005–2007,2008–2010,2011–2013,2014–2016,2017–2021).
(DOCX)

S1 Fig Cumulative incidence of cardiovascular diseases, gastric cancer, and other causes of death after gastric cancer diagnosis. (TIF)
