# Supplementary material for: Risk of cardiovascular mortality in patients with gastric cancer
Source: PLoS One. 2025 Nov 7;20(11):e0335989. doi: 10.1371/journal.pone.0335989 (PMC12594414; doi:10.1371/journal.pone.0335989)
Supplement: S1 Table — Abbreviations: CI, confidence interval; IRR, incidence-rate ratio; MR, mortality rate per 100 person-years; N, number of deaths. a IRRs were adjusted for age at follow-up (30–39 years, every 10years thereafter, and ≥80 years).sex (female or male), race (white, Black, or other), county (counties in metropolitan areas with a population larger than 1 million, counties in metropolitan areas with a population of 250,000–1,000,000, counties in metropolitan areas with a population of less than250,000,non-metropolitan counties not adjacent to a metropolitan area, or non-metropolitan counties adjacent to a metropolitan area), and calendar year at follow-up (1990–1992,1993–1995,1996–1998,1999–2001,2002–2004,2005–2007,2008–2010,2011–2013,2014–2016,2017–2021). (DOCX) [file pone.0335989.s001.docx]

**S1 Table. Incidence rate ratios (IRRs) of cardiovascular mortality in GC patients by time since cancer diagnosis, compared with the general population.**

| **By time since diagnosis** | **Patients**  **N (MR)** | **IRR (95% CI)**^a^ |
| --- | --- | --- |
| 0 to <1 month | 184 (7.15) | 11.52 (9.97-13.31) |
| 1 to <6 month | 158 (1.54) | 2.62 (2.24-3.06) |
| 6 to <12 month | 114 (1.18) | 2.10 (1.75-2.53) |
| 1 to <2 years | 131 (0.97) | 1.71 (1.44-2.03) |
| 2 to <5 years | 177 (0.74) | 1.33 (1.15-1.54) |
| 5 to <10 years | 139 (0.69) | 1.24 (1.05-1.47) |
| >10 years | 89 (0.63) | 1.15 (0.93-1.41) |

Abbreviations: CI, confidence interval; IRR, incidence-rate ratio; MR, mortality rate per 100 person-years; N, number of deaths.

^a^ IRRs were adjusted for age at follow-up (30-39 years, every 10years thereafter, and ≥80 years).sex (female or male), race (white, Black, or other), county (counties in metropolitan areas with a population larger than 1 million, counties in metropolitan areas with a population of 250,000-1,000,000, counties in metropolitan areas with a population of less than250,000,non-metropolitan counties not adjacent to a metropolitan area, or non-metropolitan counties adjacent to a metropolitan area), and calendar year at follow-up (1990-1992,1993-1995,1996-1998,1999-2001,2002-2004,2005-2007,2008-2010,2011-2013,2014-2016,2017-2021).
